# Lightweight DeepLabv3+ for Semantic Food Segmentation

**DOI:** 10.3390/foods14081306

**Published:** 2025-04-09

**Authors:** Bastián Muñoz, Angela Martínez-Arroyo, Constanza Acevedo, Eduardo Aguilar

**Affiliations:** 1 Departamento de Ingeniería y Sistemas de Computación, Universidad Católica del Norte, Av. Angamos 0610, Antofagasta 1270709, Chile; bastian.munoz01@alumnos.ucn.cl; 2 Centro de Micro-Bioinnovación (CMBi), Escuela de Nutrición y Dietética, Facultad de Farmacia, Universidad de Valparaíso, Valparaíso 2360102, Chile; angela.martinez@uv.cl; 3 Centro de Investigación del Comportamiento Alimentario (CEIC), Escuela de Nutrición y Dietética, Facultad de Farmacia, Universidad de Valparaíso, Valparaíso 2360102, Chile; constanza.acevedo@uv.cl; 4 Departament de Matemàtiques i Informàtica, Universitat de Barcelona, Gran Via de les Corts Catalanes 585, 08007 Barcelona, Spain

**Keywords:** semantic food segmentation, lightweight networks, attention mechanism, DeepLabv3+

## Abstract

Advancements in artificial intelligence, particularly in computer vision, have driven the research and development of visual food analysis systems focused primarily on enhancing people’s well-being. Food analysis can be performed at various levels of granularity, with food segmentation being a major component of numerous real-world applications. Deep learning-based methodologies have demonstrated promising results in food segmentation; however, many of these approaches demand high computational resources, making them impractical for low-performance devices. In this research, a novel, lightweight, deep learning-based method for semantic food segmentation is proposed. To achieve this, the state-of-the-art DeepLabv3+ model was adapted by optimizing the backbone with the lightweight network EfficientNet-B1, replacing the Atrous Spatial Pyramid Pooling (ASPP) in the neck with Cascade Waterfall ASPP (CWASPP), and refining the encoder output using the squeeze-and-excitation attention mechanism. To validate the method, four publicly available food datasets were selected. Additionally, a new food segmentation dataset consisting of self-acquired food images was introduced and included in the validation. The results demonstrate that high performance can be achieved at a significantly lower cost. The proposed method yields results that are either better than or comparable to those of state-of-the-art techniques while requiring significantly lower computational costs. In conclusion, this research demonstrates the potential of deep learning to perform food image segmentation on low-performance stand-alone devices, paving the way for more efficient, cost-effective, and scalable food analysis applications.

## 1. Introduction

Being aware of the nutritional value of the food we eat enables informed dietary decisions, which are crucial for individuals to receive appropriate treatment and personalized nutrition recommendations. However, measuring individuals’ dietary intake is challenging because traditional dietary assessments, such as 24-h recall, food frequency questionnaires, and food recall methods, involve random and systematic measurement errors [1]. Therefore, additional food analysis tools are essential to complement traditional dietary assessments and improve the accuracy of food intake estimation.

Promising advancements in computer vision, driven by deep learning, have inspired researchers to automate food analysis. In computer vision, food image analysis encompasses several tasks that serve various purposes, including preventing food allergies and intolerances, automating billing processes in self-service restaurants, monitoring diets, and much more [2]. Food recognition, detection, and segmentation are among the most popular tasks discussed in the literature. This research focuses on food segmentation, which is particularly valuable for monitoring dietary intake, as it not only identifies the food category but also provides food regions, enabling the estimation of the nutritional values, such as calorie content [3]. Segmenting food images is a challenging task due to the inherent characteristics of the food itself. Food can be considered a deformable or non-rigid object, meaning its visual appearance (including shape, texture, and color) can vary significantly depending on how it is cooked or served. In addition, food images often exhibit high intraclass variation and high interclass similarity, meaning that images of the same food can differ greatly, while images of different food categories can appear quite similar. Therefore, advanced computer vision models are required to effectively address the unique characteristics of food.

Improvements in the performance of deep learning models are often accompanied by higher demands for computational resources. This trend is also observed in food segmentation. For example, the method proposed in [4] achieved state-of-the-art results on the UECFoodPixComplete dataset but comes at the cost of having over 100 million parameters and 90G MACs (Multiply–Accumulate Operations). The high computational cost of such models makes them impractical for deployment on mobile or embedded devices. Typically, the solution is to implement the model on an external server and connect to its services via an API [5]. However, this approach introduces maintenance costs and requires an internet connection for access. Unlike traditional deep learning methods, research on lightweight deep networks focuses on reducing the computational complexity of large models by optimizing their design or applying model compression techniques without significantly decreasing performance [6]. Therefore, lightweight deep networks become promising candidates for providing real-time solutions in standalone mobile applications.

A limited number of lightweight methods have been introduced for food-related tasks, mainly in food recognition [7,8] and calorie estimation [9,10]. Regarding semantic food segmentation, to the best of our knowledge, only one method has been proposed. Mid-DeepLabv3+ [11] is a lightweight variation of the DeepLabv3+ [12] architecture. The backbone of the original DeepLabv3+ is optimized by using a reduced version of ResNet50 [13], which excludes the last convolution block. To mitigate performance loss, a new middle layer is added to the decoder path, along with a SimAM [14] attention mechanism. The additional layer helps recover features potentially lost in the encoder, while the attention mechanism further improves performance.

Our work also proposes a lightweight variant of DeepLabv3+, although the optimization differs from the previous approach. The Mid-DeepLabv3+ optimization focuses on the backbone, simplifying it and compensating for the loss in feature representation with an intermediate layer (middle-level feature) that feeds into the decoder. In addition, the low-, middle-, and high-level features extracted from the backbone are passed through an attention mechanism for refinement. Unlike Mid-DeepLabv3+, the proposed method does not simplify a deep network but instead employs an efficient network as a backbone, reducing computational costs without compromising feature representation capability. Additionally, in the neck of the encoder, multi-scale feature extraction is optimized by significantly reducing parameters and MACs, while the refined high-level features are further improved through the incorporation of an attention mechanism in the encoder head.

The main contributions of this work include optimizing the backbone, neck, and head of the DeepLabv3+ encoder to propose a lightweight deep learning method for semantic food segmentation. Additionally, the first dataset for food image segmentation focused on the Chilean diet is introduced. This dataset includes segmentation masks and food composition data for self-acquired images of the most commonly consumed food categories among university students. Furthermore, the proposed ILW-DeepLabv3+ method is compared with state-of-the-art methods on five public datasets, achieving competitive or even superior results while significantly reducing computational costs. Finally, a qualitative analysis of the results is conducted by considering the predictive uncertainty estimated through a conformal learning [15] post hoc approach.

This work extends our previous conference paper [16] by improving the lightweight DeepLabv3+ method with an attention mechanism. We also conduct additional performance evaluations on three new datasets: CamerFood10 [11], MyFood [17], and SAI-ChileanFoodSeg, the latter of which was proposed by us and includes both segmentation and food composition data. Furthermore, we apply a conformal learning method as a post hoc strategy to extract the predictive uncertainty in the segmented masks and perform a qualitative analysis of the results based on it. Finally, the results demonstrate the effectiveness of our approach, demonstrating competitive performance with improved mIoU compared to the state of the art, while requiring significantly fewer computational resources.

## 2. Related Works

### 2.1. Lightweight Deep Learning

Recent advancements in Convolutional Neural Networks (CNNs) have focused on designing lightweight architectures, which are essential for deployment on resource-constrained devices such as mobile phones or embedded systems.

Minimizing computational demands is crucial, as it reduces training times, decreases energy consumption, and enhances model efficiency for real-world deployment. Running models directly on mobile devices in offline mode eliminates the need for a constant internet connection [18,19], making it a more practical alternative to a client–server approach, which requires high server maintenance costs and stable network connectivity. This reliance on cloud-based processing excludes remote areas, such as rural regions, where network coverage is often unreliable or unavailable [20]. To address these challenges, several lightweight CNN architectures have been developed to optimize efficiency and performance in resource-constrained environments, with MobileNetV3 [21], EfficientNet [22], and ShuffleNet [23] being used frequently.

The development of lightweight models has also facilitated food recognition and calorie estimation on resource-limited devices. Haque et al. [9] addressed the need for real-time calorie estimation by introducing a parameter-optimized CNN capable of running on handheld devices without relying on external servers. Sajith et al. [10] demonstrated the effectiveness of MobileNetV2 for nutrient estimation, achieving high accuracy and usability in a mobile diet management application. Sheng et al. [7] proposed a technique combining transformer grouping and token shuffling, striking a balance between computational efficiency and recognition accuracy. More recently, Sheng et al. [8] refined this concept by introducing the Efficient Hybrid Food Recognition Network (EHFR-Net), which integrates CNNs with vision transformers to preserve spatial information, ultimately improving food recognition accuracy.

### 2.2. Semantic Segmentation

Image segmentation is a fundamental task in computer vision that involves partitioning an image into meaningful regions for further analysis. Among the various segmentation techniques, semantic segmentation has gained significant attention as it assigns a class label to each pixel, enabling a detailed and structured understanding of an image at the pixel level [12,24].

Several methods have been proposed for food image segmentation. Aslan et al. [25,26] conducted extensive research on automatic diet monitoring, utilizing networks like DeepLab and SegNet to differentiate between food and non-food items in segmentation tasks. In [27], DeepLabv3+ with default parameters was used to establish baseline performance on a new segmentation dataset. A comparison between DeepLabv3+ and a modified version of YOLACT for semantic segmentation was conducted in [28], demonstrating an improvement of about 1% in YOLACT on the proposed dataset. Additionally, CDPN [29] and FDSNet [4] were introduced to enhance semantic food segmentation for dietary monitoring applications. CDPN employs convolutional and deconvolutional layers to construct a feature pyramid, enabling dense and precise segmentation maps with strong multi-scale representation. On the other hand, FDSNet integrates a Swin Transformer and CNNs within a dual-branch architecture, incorporating a multi-scale relation-aware feature fusion module to enhance segmentation across different resolutions.

Attention mechanisms have been integrated into food image segmentation methods to improve efficiency and accuracy while maintaining computational costs. GourmetNet [30] improves segmentation by using Waterfall Atrous Spatial Pooling (WASPv2) and integrating both spatial and channel attention modules to refine multi-scale feature extraction. CANet [31] introduces a cross-spatial attention mechanism that captures long-range dependencies along with a channel attention module to improve feature representation and accuracy. Additionally, BayesianGourmetNet [32] extends both DeepLabv3+ and GourmetNet by incorporating Bayesian deep learning with MC-Dropout to estimate uncertainty, thereby improving reliability in segmentation prediction. Finally, closer to our work, Mid-DeepLabv3+ [11] was introduced as a lightweight approach to food segmentation, in which DeepLabv3+ was modified by integrating an additional middle feature refinement layer and incorporating the SIMAM attention mechanism for the last, middle, and lower layers.

These models illustrate the diverse approaches to improve food segmentation, including multi-scale feature extraction, attention mechanisms, and uncertainty modeling, demonstrating ongoing efforts to refine accuracy and generalization across various datasets.

## 3. Materials and Methods

### 3.1. SAI-ChileanFoodSeg Dataset

This dataset was created from the multi-label food recognition dataset, SAI-ChileanDiet [33], which contains images collected from two sources: Google Images and images taken by university students. We used all the images provided by the students, annotating each with segmentation masks and the corresponding food composition data.

#### 3.1.1. Segmentation Mask Labeling

The annotation process was carried out by three external annotators, who meticulously labeled each image using the Labelbox platform (https://labelbox.com/, accessed on 17 February 2025). In addition to the images, the annotators were provided with the original multi-label annotations for each image, labeling the segmentation masks only for these foods. Over three months, 1216 images were labeled, covering a total of 102 foods. As a result of this process, a JSON file was generated for each image, containing the image name, the food names, the color assigned to each food, and the URL linking to the image with its corresponding segmentation mask. Once the annotations were completed, taking into account the data provided by all annotators, the segmentation masks were reconstructed by consolidating the colors assigned to each food. Afterward, the research team manually reviewed the segmentation masks related to each image and corrected any discrepancies between annotators. Additionally, the images associated with each food were counted, and foods with fewer than 5 images were categorized as *other food*, reducing the number of food categories from 102 to 71. Finally, the segmentation masks assigned to images categorized solely as *other food* were removed, leaving a total of 1177 images.

#### 3.1.2. Food Composition Estimation

A trained dietitian (a postgraduate student) compared participants’ food or recipe (mixed-dish) images with information in the photographic Atlas of Chilean Foods and Typical Preparations [34]. This atlas is a Chilean government document used in the last National Food Consumption Survey 2010 [35] and contains images of food, beverages, and Chilean recipes, along with ingredient information, including the number of grams, for each image. For food images and recipes not found in the atlas, the dietitian searched for similar images on Chilean supermarket websites to estimate food serving sizes and convert them into metric units (milliliters and grams). Since we used images of students’ real-life recipes or mixed dishes, some ingredients were difficult to identify. For example, in an image of a Chilean hot dog, we could only identify the bread and sauces. However, Chilean hot dogs contain a sausage, avocado, and tomato. Dietitians also considered yield and retention factors to estimate the weight of the food and recipes in the images [36].

After estimating the number of grams of each food and/or ingredient in the images, the nutritionist calculated the food composition values (e.g., energy, protein, carbohydrates, fats, and sodium) using the proportionality method with the Chilean Food Composition Table [37] and information from Chilean e-commerce companies (i.e., nutritional labels). The missing food composition values were sourced from the US Department of Agriculture [38]. Oil and salt were excluded due to imprecise quantities in the recipes. Dietitians also considered yield and retention factors to estimate the nutrient composition of cooked foods [36,39].

#### 3.1.3. Data Statistics

**Figure 1:**
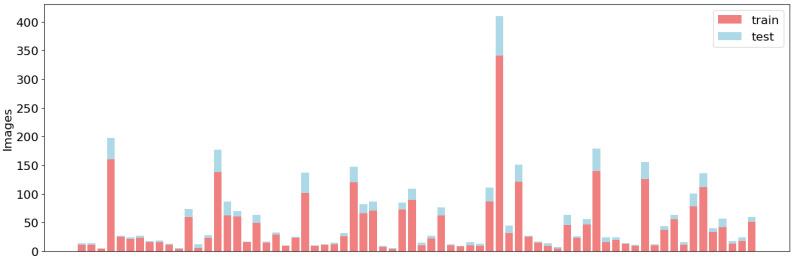
Data distribution for the training and test sets of the proposed SAI-ChileanFoodSeg dataset. The y-axis corresponds to the number of images and the bars correspond to the class labels.

The resulting SIA-ChileanSeg dataset contained 1177 images with pixel-wise segmentation annotations for 71 foods and the corresponding food composition data. The dataset was divided into two groups: 80% of the images were used for training and the remaining 20% were used for testing. As shown in Figure 1, the division of the training and test sets maintained a similar distribution of food categories. However, an imbalance in the data is evident across all sets.

### 3.2. Improved LW-DeepLabv3+

**Figure 2:**
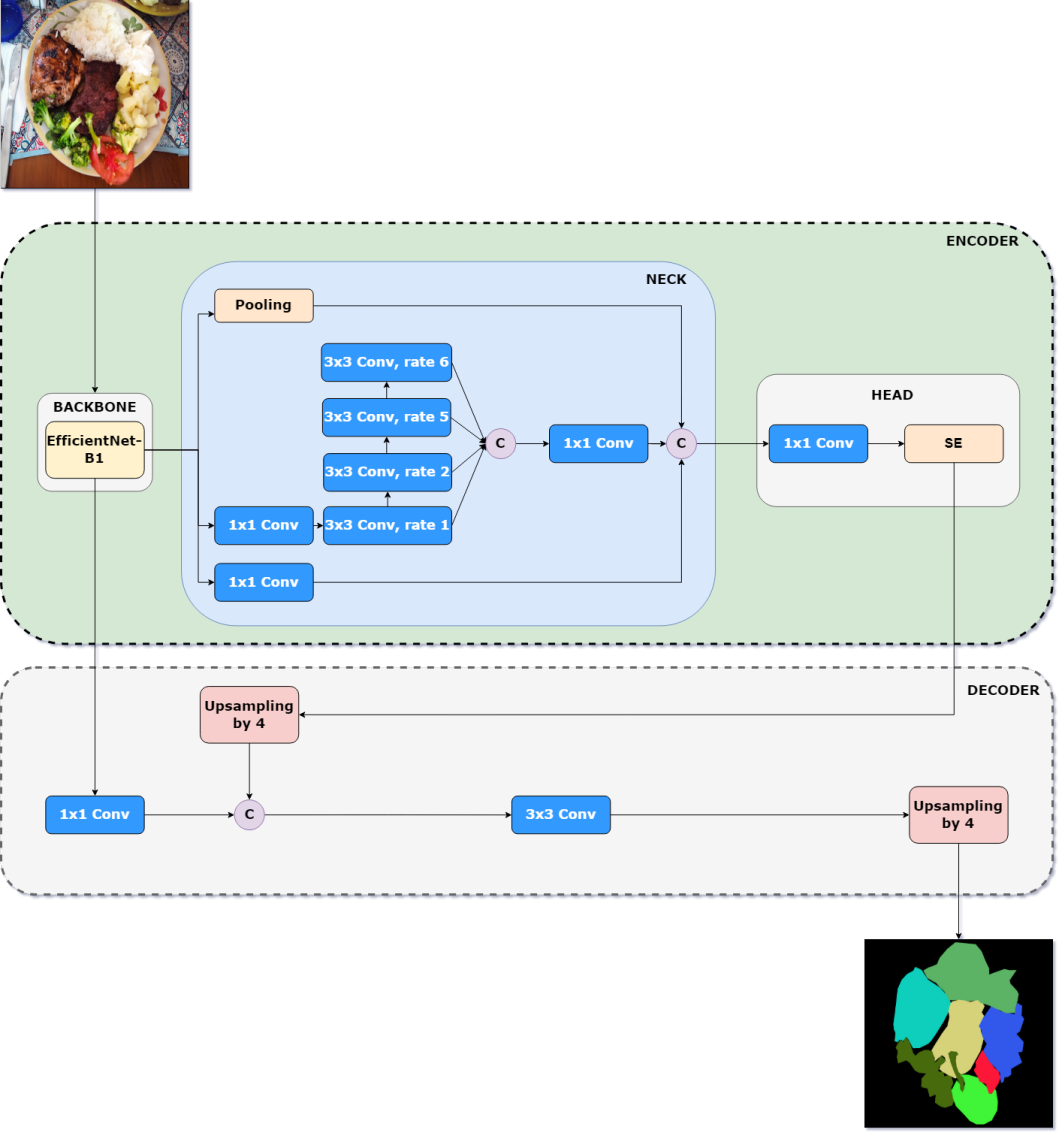
Pipeline of the improved LW-DeepLabv3+ method for semantic food segmentation. **C** (purple circle) stands for the concatenation operation and **rate** is the dilation factor of the convolutional filter.

In our previous work, we proposed optimizing DeepLabv3+ for efficient semantic segmentation in food images and introduced a lightweight variant, named LW-DeepLabv3+ [16]. In this variant, the original DeepLabv3+ backbone was replaced with EfficientNet-B1, a lightweight model that optimally balances depth, width, and resolution to maximize accuracy without significantly increasing computational cost. Additionally, the ASPP module was replaced with Cascade Waterfall ASPP (CWASPP), a design that organizes atrous convolutions in a cascade rather than in parallel, allowing for the progressive integration of extracted features and reducing the number of parameters and operations required. In this work, we further improve the performance of LW-DeepLabv3+ by incorporating an attention mechanism, resulting in a minimal increase in model complexity. An overview of the improved LW-DeepLabv3+ (ILW-DeepLabv3+) is shown in Figure 2.

In the following subsections, the details of the architecture are presented, and the key components and enhancements that contribute to its efficiency and segmentation accuracy are outlined.

#### 3.2.1. Encoder: Backbone

The original backbone in DeepLabv3+ is Xception, selected for its efficiency and strong feature extraction capabilities. Xception (Extreme Inception) is a deep convolutional architecture that extends the Inception model by replacing standard convolutions with depthwise separable convolutions, significantly reducing computational complexity while maintaining high performance. The Xception architecture is composed of three main stages: entry flow, middle flow, and exit flow, where each block consists of depthwise separable convolutions, followed by batch normalization and ReLU activation. Unlike traditional convolutional networks, Xception decouples spatial and channel-wise feature extraction, enabling more efficient feature representation. In DeepLabv3+, Xception was further optimized for semantic segmentation by removing max pooling operations and replacing them with strided separable convolutions, allowing the model to extract denser feature maps. Additionally, atrous separable convolutions were applied in both the ASPP module and the decoder, enhancing multi-scale feature extraction while maintaining computational efficiency.

To improve efficiency while maintaining segmentation accuracy, several backbones were evaluated for DeepLabv3+ [16,40]. EfficientNet-B1 [22] was identified as providing an optimal balance between precision and computational complexity. Therefore, we replaced Xception with EfficientNet-B1, which is part of a family of models that employs a composite scaling approach, jointly adjusting depth, width, and resolution to optimize performance. Unlike traditional models that scale only one dimension (such as increasing depth in ResNets or width in WideResNets), EfficientNet-B1 utilizes a compound coefficient to scale all three dimensions in a balanced manner, ensuring an optimal accuracy-to-efficiency trade-off. This strategy allows the model to achieve high segmentation accuracy without disproportionately increasing computational resource consumption, making it a suitable alternative for lightweight semantic segmentation tasks.

The EfficientNet architecture is based on Mobile Inverted Bottleneck Convolutions (MBConv) [41], which enhance feature extraction while maintaining a low parameter count. It also incorporates squeeze-and-excitation (SE) blocks, which improve channel-wise feature recalibration, thereby increasing robustness to variations in input images. Furthermore, EfficientNet-B1 utilizes depthwise separable convolutions, which reduce computational complexity while maintaining high efficiency.

#### 3.2.2. Encoder: Neck

The Atrous Spatial Pyramid Pooling (ASPP) module is a key component of DeepLabv3+ and is designed to capture multi-scale contextual information by applying multiple parallel atrous convolutions with different dilation rates. In standard implementations, ASPP typically employs three atrous convolutions with dilation rates of 6, 12, and 18, along with a 1 × 1 convolution and a global average pooling layer. This multi-branch structure enables the network to aggregate features at various receptive fields, improving segmentation performance. However, one of the main challenges of ASPP is the grid effect, which arises when large dilation rates cause missing spatial details due to uneven sampling across the feature map. This issue can lead to information loss and suboptimal segmentation, particularly in fine-grained structures.

In the proposed method, the ASPP module is replaced with the Cascade Waterfall ASPP (CWASPP) module [42], an optimized version of the ASPP block used in DeepLabv3+, offering a more efficient and accurate solution. Its name derives from the cascading structure, along with the use of atrous convolutions, which facilitates the capturing of details at different spatial scales. Unlike the standard ASPP module, where atrous convolutions operate in parallel, CWASPP organizes these operations sequentially, with each layer receiving the output from the previous one. This enables a more progressive and efficient integration of the extracted features. Additionally, a 1 × 1 convolution is applied at the input to reduce the number of parameters. In contrast to ASPP, which often uses larger dilation rates that can cause the grid effect and lead to information loss, CWASPP employs a progressive approach with smaller dilation rates, effectively avoiding the grid effect.

#### 3.2.3. Encoder: Head

To further improve the performance of the proposed lightweight DeepLabv3+ method without increasing computational complexity, an attention mechanism (AM) was added. In [43], the AM was added to the head of the encoder. However, in our method, it is applied after the 1 × 1 convolution layer for greater efficiency.

Several state-of-the-art AMs [14,44,45,46,47,48] were evaluated (see Section 5.1), with SE [46] delivering the most stable results across all the target datasets in our experiments. The SE block is designed to improve feature representation by modeling interdependencies between channels. It works by applying a squeeze operation, which performs global average pooling to condense spatial information. This is followed by an excitation operation, where a lightweight two-layer fully connected network learns channel-wise attention weights. The output is a recalibrated feature map where each channel is weighted according to its importance.

#### 3.2.4. Decoder

The original decoder of DeepLabv3+ is used in the proposed method, preserving its design for refining segmentation boundaries and enhancing spatial details. The decoder operates by first extracting a low-level feature map from the output layer of EfficientNet-B1. This feature is then processed through a 1 × 1 convolution to reduce the number of channels while maintaining spatial resolution. Meanwhile, the high-level feature map is processed using the CWASPP module, which applies cascaded atrous convolutions to capture multi-scale contextual information more efficiently. The output of CWASPP is then refined with a 1 × 1 convolution before being enhanced by the SE module, which adaptively recalibrates channel-wise feature responses by modeling interdependencies between channels. This processed feature map is then upsampled by a factor of 4 and concatenated with the 1 × 1 convolution of the low-level feature map. The fused representation is subsequently passed through a 3 × 3 convolution, enhancing feature discrimination and segmentation accuracy. Finally, the output undergoes a final upsampling by a factor of 4, restoring the resolution to match the original input size and generating the final segmentation mask. This approach ensures the effective integration of multi-scale features while leveraging attention mechanisms to improve segmentation performance.

## 4. Validation

### 4.1. Food Datasets

The following food segmentation datasets were selected to validate the proposed ILW-DeepLabv3+ method, ensuring a comprehensive evaluation across diverse food types and imaging conditions and providing a robust benchmark for assessing the accuracy and efficiency of the models:

- **UECFoodPixComplete** [27] is a Japanese food dataset containing 10,000 images and 103 food categories. It is split into 9000 images for training and 1000 for testing. The dataset underwent rigorous manual pixel-wise annotations, resulting in high-quality ground-truth (GT) masks.
- **UNIMIB2016** [49] is an Italian food dataset comprising 1027 images and 73 food categories. The dataset features manually segmented images, where polygonal boundaries were carefully annotated to ensure high precision. Captured on canteen trays under semi-controlled conditions, it is a valuable benchmark for training and evaluating semantic segmentation models, enhancing their applicability in real-world scenarios. The dataset is divided into 650 images for training and 360 for testing.
- **MyFood** [17] is a food segmentation and classification dataset designed to support nutritional monitoring. It consists of 1250 images, with 1000 images for training and 250 for testing, distributed across 10 food categories, representing some of the most commonly consumed foods in Brazil. The dataset was created by collecting images from Google Images, Flickr, and the Bing API and manually annotated using polygon masks with the VGG Image Annotator (VIA) tool.
- **CamerFood10** [11] is a newly introduced food segmentation dataset specifically focused on Cameroonian cuisine. It consists of 1242 images, with 1032 images for training and 210 for testing. The dataset was created by collecting images from search engines and social media platforms, followed by a rigorous cleaning and annotation process using polygon masks. It features 10 of the most commonly consumed food categories in Cameroon, aiming to address the lack of publicly available datasets for African food segmentation.

### 4.2. Experimental Settings

The proposed method was trained for 100 epochs with a batch size of 8. Cross-entropy loss was used as the loss function, and optimization was carried out using the Stochastic Gradient Descent (SGD) optimizer. To enhance optimization stability, a momentum of 0.9 and a weight decay of 0.0001 were applied. The learning rate was set to 0.01 for the UECFoodPixComplete dataset and 0.05 for the other datasets, with a polynomial decay schedule at a power of 0.9.

For image processing, all input images were resized to 512 × 512 pixels, and pixel values were scaled to the range from 0 to 1. As a data augmentation strategy, a random horizontal flip was applied during training. Regarding computational resources, the experiments were conducted using the PyTorch 2.4.1 framework on a server with an NVIDIA GeForce RTX 4090 (24 GB) GPU (Santa Clara, CA, USA), a 13th-generation Intel Core i9-13900K processor (Santa Clara, CA, USA), and 62 GB of RAM. For inference time estimation (GPU and CPU), a server with an NVIDIA GeForce RTX 3060 (12 GB) (Santa Clara, CA, USA) and an AMD Ryzen 5 5600X processor (Santa Clara, CA, USA) was used to better highlight the differences between the models.

### 4.3. Evaluation Metrics

One of the most commonly used metrics in semantic segmentation tasks is the Mean Intersection over Union (mIoU), which measures the overlap between the predicted and actual regions in the image. This metric is formally defined as

(1) $$mIoU=\frac{1}{N}\sum_{i=1}^{N}\frac{1}{K}\sum_{k=1}^{K}\frac{|y_{i,k}\cap {\hat{y}}_{i,k}|}{|y_{i,k}\cup {\hat{y}}_{i,k}|}$$

where *N* is the total number of images and *K* is the number of classes. The terms $y_{i,k}$ and ${\hat{y}}_{i,k}$ denote the GT and predicted segmentation masks for class *k* in image *i*.

## 5. Results

This section details the results of an extensive evaluation of the proposed model using the UECFoodPixComplete, UNIMIB2016, MyFood, CamerFood10, and SAI-ChileanFoodSeg datasets to measure its effectiveness in semantic food segmentation. Performance is analyzed based on the mIoU metric, with additional comparisons considering model complexity, including parameter count and MACs, to assess computational efficiency.

### 5.1. Performance Using Different Attention Mechanisms

The effectiveness of integrating the attention mechanism into ILW-DeepLabv3+ is shown in Table 1, which shows consistent improvements across all datasets. The baseline model without an attention mechanism achieved the lowest segmentation performance in terms of mIoU among all datasets. The inclusion of various attention mechanisms, such as CBAM, SE, GAN, TAN, ECA, and SIMAM, resulted in notable performance gains, thereby confirming their role in enhancing feature representation. Among them, GAM achieved the highest mIoU values of 69.85% and 80.40% on UECFoodPixComplete and UNIMIB2016, respectively, whereas SE achieved the highest mIoU values of 84.51% and 84.69% on MyFood and CamerFood10, respectively.

To analyze the trade-off between accuracy and computational efficiency, the parameter and GMAC variations introduced by these mechanisms are shown in Table 2. The baseline (LW-DeepLabv3+) consists of 6.79M parameters and 2541M MACs, with each attention mechanism introducing minor parameter increments. CBAM and SE added approximately 8k parameters each, while GAM increased the count by 32k, resulting in an additional 33.67M MACs. In contrast, ECA and SIMAM resulted in improvements with almost no increase in parameters 5 and 0, respectively, making them good choices for maintaining a lightweight model, provided the mIoU loss is acceptable. These results demonstrate that incorporating attention mechanisms can significantly improve segmentation accuracy while adding minimal computational cost, making them valuable for efficient food segmentation models in resource-constrained environments.

**Figure 3:**
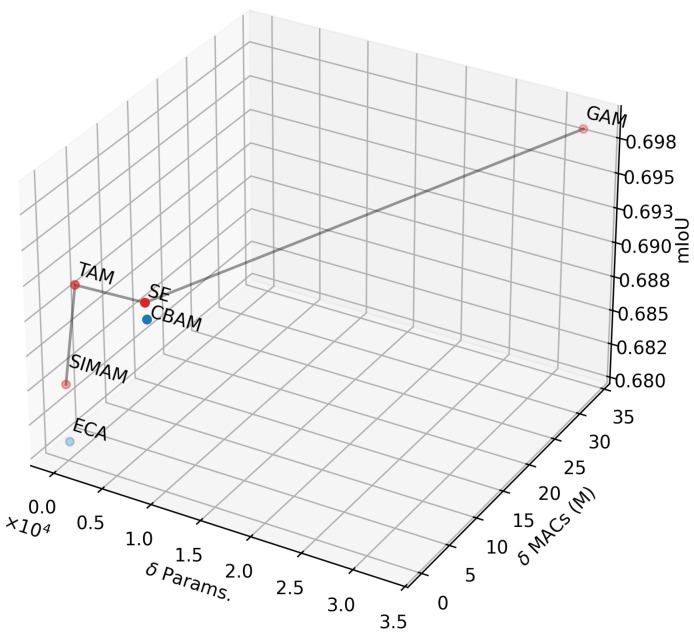
Pareto frontier for three optimization objectives: parameter minimization, MAC minimization, and mIoU maximization.

Figure 3 illustrates the Pareto frontier after optimizing the computational efficiency (Params. and MACs) and segmentation accuracy (mIoU) of the proposed method by varying the attention mechanism. Four attention mechanisms are highlighted on the Pareto frontier (in red) as optimal solutions: SIMAM, TAM, SE, and GAM. Among them, TAM and SE offer the best trade-offs between Params., MACs, and mIoU. By comparing the segmentation accuracy of TAM and SE, it was observed that SE consistently achieved stable performance across nearly all datasets (see Table 1). Therefore, SE was selected for inclusion in the proposed method.

### 5.2. Impact of Learning Rate on Model Training

The proposed ILW-DeepLabv3+ method was trained on all datasets using the same hyperparameters, except for the learning rate. The reason for this was the nature of the target datasets, particularly in terms of the number of images in each. Note that during training, the learning rate was reduced using a polynomial decay after each iteration until it reached 0 when training was complete. Therefore, if there is limited data, it is highly likely that the model will underfit, as the learning rate rapidly decreases to 0. Since the UECFoodComplete dataset contains approximately 10 times more images than the other datasets, we decided to set the learning rate for the remaining datasets to be 5 times the value used in the UECFoodComplete experiments.

Two datasets were selected to illustrate the impact of the learning rate on model training: UECFoodPixComplete, with 9000 training images, and UNIMIB2016, with 650 training images. The results are shown in Table 3. In the case of UECFoodPixComplete, as expected, a higher learning rate tended to result in overfitting, while a lower rate led to underfitting. A learning rate of 0.10 yielded the best performance. In the case of UNIMIB2016, strong underfitting was observed with a learning rate of 0.001, highlighting the rapid decay of the learning rate. As the learning rate increased, performance improved accordingly. The improvement from 0.05 to 0.1 was less pronounced than that from 0.01 to 0.05, indicating that the learning rate approached its optimal value before the model started to overfit. Although the best learning rate for UNIMIB2016 was 0.1, a learning rate of 0.05 was used for the other datasets, aside from UECFoodPixComplete, to mitigate the risk of overfitting.

### 5.3. Ablation Study

The results shown in Table 4 provide a clearer understanding of the effect of each optimization on model performance, accuracy (mIoU), and computational complexity. Notably, optimizing the backbone had the greatest impact, significantly reducing the number of parameters and MACs, halving the inference time on both the GPU and CPU, and increasing the mIoU by more than 2%. Optimizing the neck further improved parameters, MACs, and inference time but at the cost of model accuracy. Optimizing the head mitigated this issue by maintaining computational cost while improving performance by more than 0.5% compared to the second-best result.

Additionally, the performance of the proposed method was assessed using different lightweight backbones. Table 5 presents the results for ILW-DeepLabv3+ when the EfficientNet-B1 backbone was replaced with ShuffleNetV2+ or MobileNetv3-L. Although ShuffleNetV2+ resulted in the fastest prediction, a performance decrease of more than 40% was observed when using it as the backbone, significantly affecting the quality of the segmentation results. On the other hand, MobileNetv3-L can be considered a good alternative if computational efficiency needs to be prioritized, as it yielded inference times twice as fast as those of EfficientNet-B1 but lost about 8% and 4.5% in the mIoU on the UECFoodPixComplete and CamerFood10 datasets. The performance difference between lighter alternatives and EfficientNet-B1 was also more pronounced on large-scale datasets, highlighting that EfficientNet-B1 is a better option for scalable solutions.

### 5.4. Semantic Food Segmentation Benchmark

The proposed ILW-DeepLabv3+ was evaluated and compared against state-of-the-art models across multiple food datasets. The results are presented in Table 6, Table 7, Table 8 and Table 9 for the test sets from the UECFoodPixComplete, UNIMIB2016, MyFood, and CamerFood10 datasets, respectively.

As shown in Table 6, the results on the UECFoodPixComplete dataset highlight the efficiency of the proposed ILW-DeepLabv3+ method, which achieved an mIoU of 69.26%, surpassing our previous LW-DeepLabv3+ method by more than 4% while maintaining a low computational cost of 2.54G MACs and 6.80M parameters. ILW-DeepLabv3+ outperformed heavier alternatives such as DeepLabv3+, with 45.70M parameters and 57.12G MACs, and FoodSAM, with 636M parameters and 1016.72G MACs. FDSNET achieved the best mIoU of 75.89% but at a much higher cost of 101.93M parameters and 91.37G MACs, making it impractical for resource-limited applications. CANet, with an mIoU of 68.90%, also had high computational requirements, with 40.10M parameters and 127.72G MACs.

Table 7 demonstrates that on the UNIMIB2016 dataset, ILW-DeepLabv3+ achieved an mIoU of 79.18%, surpassing LW-DeepLabv3+, which reached 74.49%. Despite its superior performance, ILW-DeepLabv3+ remained lightweight, with only 6.79M parameters and 2.34G MACs. In comparison, BayesianGourmetNet achieved the best performance but required significantly more resources, with 91.79M parameters and 4.73G MACs, making ILW-DeepLabv3+ the more efficient option.

Table 8 confirms this trend on the MyFood dataset, where ILW-DeepLabv3+ achieved the best performance with an mIoU of 84.51%, outperforming the second-best model, CDPN, by over 7%. Additionally, it utilized fewer resources, with only 6.77M parameters and 1.89G MACs.

A similar pattern was observed on the CamerFood10 dataset (see Table 9), where ILW-DeepLabv3+ achieved an mIoU of 84.69%, outperforming Mid-DeepLabv3+ by approximately 20% while using about 30% fewer parameters and 95% fewer MACs. This further reinforces the effectiveness of the proposed method for semantic food segmentation.

Regarding inference times, Table 6, Table 7, Table 8 and Table 9 show that the proposed ILW-DeepLabv3+ is at least 30% faster than the state-of-the-art across all datasets when using the CPU, highlighting the effectiveness of the optimizations. This results in a method that requires fewer parameters and MACs and yields the best (or second-best) segmentation accuracy.

### 5.5. Qualitative Analysis of the Segmentation Results

**Figure 4:**
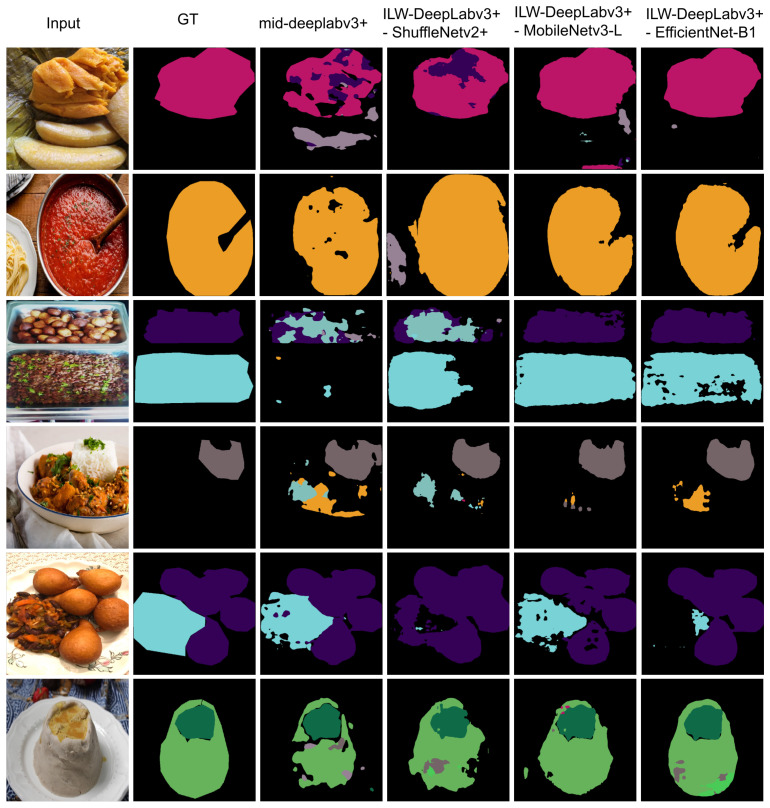
Examples of results from lightweight semantic food segmentation methods on the CamerFood10 dataset. GT denotes the ground-truth mask.

Figure 4 presents examples of the segmentation results obtained using the proposed ILW-DeepLabv3+ method with various lightweight backbones (see Table 5) and the baseline method on the CamerFood10 dataset (see Table 9). The results demonstrate that the proposed method with either MobileNetv3-L or EfficientNet-B1 significantly outperforms Mid-DeepLabv3+. Improvements are evident in the reduction in errors between different food categories and the reduction in segmented background pixels as food.

**Figure 5:**
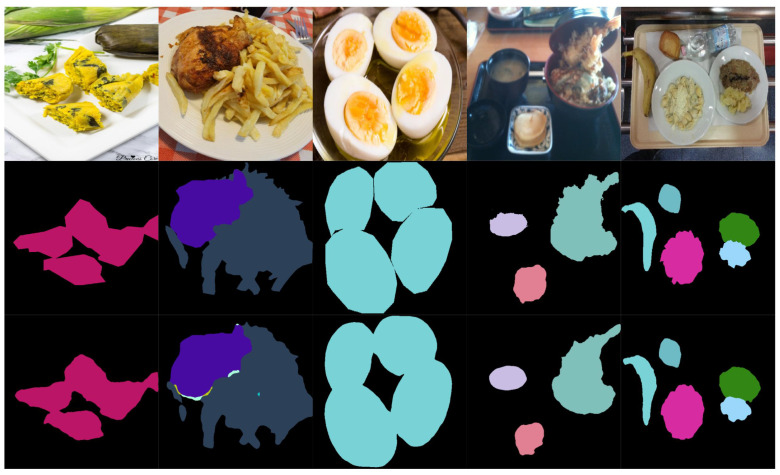
Examples of successful predictions. From left to right: the CamerFood10, SAI-ChileanFoodSeg, MyFood, UECFoodPixComplete, and UNIMIB2016 datasets. From top to bottom: the input image, GT mask, and predicted mask generated by ILW-DeepLabv3+.

**Figure 6:**
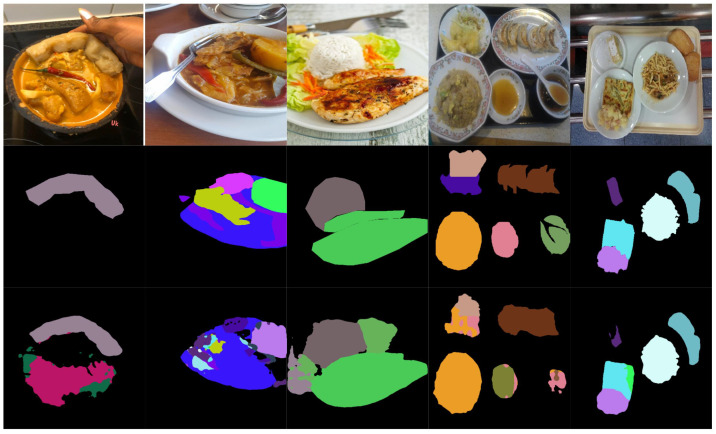
Examples of failed predictions. From left to right: the CamerFood10, SAI-ChileanFoodSeg, MyFood, UECFoodPixComplete, and UNIMIB2016 datasets. From top to bottom: the input image, GT mask, and predicted mask generated by ILW-DeepLabv3+.

Figure 5 and Figure 6 illustrate examples of both successful and failed predictions using the proposed ILW-DeepLabv3+ method. The first row in each figure displays the original food images, showcasing a variety of dishes. The second row presents the GT, where manually annotated segmentation masks are used to assign distinct colors to different food items. The third row shows the predictions made by ILW-DeepLabv3+, enabling a direct comparison between the expected and generated segmentations.

In the examples of successful predictions shown in Figure 5, the proposed method demonstrates high segmentation accuracy, effectively distinguishing between food components across various dish presentations. For well-defined dishes, such as boiled eggs and meal trays, the segmentations closely match the GT, accurately capturing object boundaries and maintaining a clear differentiation between items. For more complex meals, ILW-DeepLabv3+ successfully separates overlapping ingredients, preserving both shape and spatial consistency.

On the other hand, the examples of failed predictions shown in Figure 6 highlight challenges in handling overlapping, occluded, and fluid food items. In dishes such as soups and stews, the model struggles with boundary delineation, leading to merged or fragmented regions. Similarly, in tray-based meals with multiple food components, segmentation inconsistencies occur, in which visually similar items are misclassified or incorrectly grouped. Some regions also exhibit shape distortions, particularly when items are occluded by tableware or presented in irregular orientations. These challenges emphasize areas for improvement, particularly in boundary refinement, spatial context awareness, and the handling of occluded or ambiguous food items.

**Figure 7:**
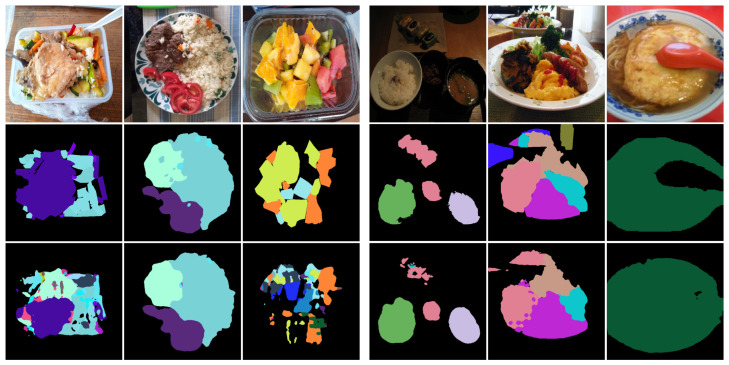
Examples of mispredictions in challenging scenarios. The images on the left correspond to the SAI-ChileanFoodSeg dataset, while those on the right correspond to the UECFoodPixComplete dataset. From top to bottom: the input image, GT mask, and predicted mask generated by ILW-DeepLabv3+.

More mispredictions can be observed in Figure 7, which presents examples of mispredictions on challenging images from the UECFoodPixComplete and SAI-ChileanFoodSeg datasets. The results highlight that segmenting small ingredients (first column) or small ingredients (e.g., rice) overlapping with others (second column) is more challenging. Additional segmentation failures occur with underrepresented classes (third column), objects that are far from the camera (fourth and fifth columns), or food that is partially obscured by tableware (sixth column). Interestingly, segmentation quality is not significantly affected by lighting conditions (fourth column).

### 5.6. Qualitative Analysis Based on Uncertainty Quantification via Conformal Prediction

Achieving perfect performance is not possible in an uncontrolled real-life environment. For this reason, it is not only important to provide the model prediction but also to estimate the confidence that the model has in that prediction, to be able to take action if necessary. For instance, in the context of semantic food segmentation, if certain regions exhibit high uncertainty, users can make corrections before utilizing them for nutrition estimation. For this purpose, a lightweight post hoc method based on conformal prediction was used to quantify predictive uncertainty [15]. Uncertainty is quantified by treating the output of the segmentation models as a multi-label segmentation mask ($\hat{Z}$), defined as follows:

$$\hat{Z}=\left\{{\hat{z}}_{ijk}:ij\in I_{HW},k\in L\right\}$$

where L={1,2,…,K} is the set of labels (or food categories) and $I_{HW}=\left\{1,\ldots ,H\right\}\times \left\{1,\ldots ,W\right\}$ represents the set of pixel indices in an image with *H* pixels of height and *W* pixels of width. The term ${\left({\hat{z}}_{ijk}\right)}_{k=1}^{K}\in {\left[0,1\right]}^{K}$ encodes the subset of predictions corresponding to the pixel ij obtained after applying the softmax function to the output layer.

Then, a coverage parameter λ∈[0,1] is used to select the appropriate labels for each pixel. Each pixel ${\hat{z}}_{ijk}$ with a softmax score above the threshold $\hat{\lambda }=1-\lambda$ is assigned a value equal to 1, and 0 otherwise. Afterward, the *K* dimensions (one for each label) of $\hat{Z}$ can be interpreted as binary segmentation masks (class *k* vs. others).

Finally, uncertainty is quantified using the VariASCo uncertainty heatmap (*V*). To do this, the number of labels for each pixel ($NL_{ij}$) is counted:

$$NL_{ij}=\left|\left\{{\hat{z}}_{ijk}:{\hat{z}}_{ijk}=1,{\hat{z}}_{ijk}\in \hat{Z}\right\}\right|$$

where |.| denotes the cardinality of the set. Then, *V* is created by dividing each pixel value ${\hat{z}}_{ijk}$ by the corresponding number of labels $NL_{ijk}$, resulting in a scalar value in the range [0,1] for each pixel.

$$V=\{\frac{{\hat{z}}_{ijk}}{NL_{ij}}:ij\in I_{HW},{\hat{z}}_{ijk}\in \hat{Z}\;\}$$

To estimate the appropriate value of $\hat{\lambda }$, a calibration set is required. In our case, we empirically set $\hat{\lambda }=0.01$.

**Figure 8:**
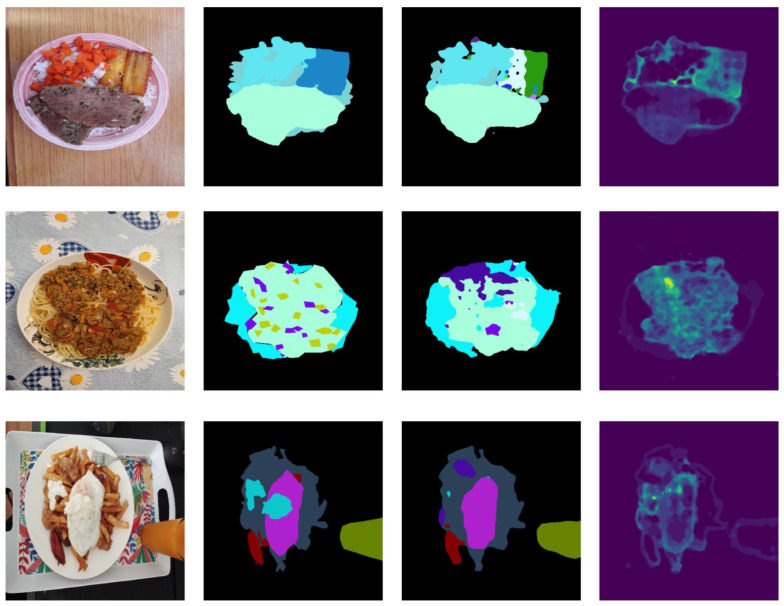
Example results of ILW-DeepLabv3+ on the SAI-ChileanFoodSeg dataset. From left to right: the input image, GT segmentation mask, predicted segmentation mask, and VariASCo uncertainty heatmap.

Figure 8 shows the predicted mask and associated uncertainty for a subset of images from the SAI-ChileanFoodSeg dataset. When examining predictive uncertainty, we observe that high uncertainty frequently occurs at the boundaries between food and background categories. Additionally, in most cases, uncertainty is high in areas where mispredictions occur, such as the cooked banana in the first row and the small vegetables over the tomato sauce in the second row. These findings highlight the importance of quantifying uncertainty to interpret the results and offer insights on how to improve the model (e.g., by incorporating more training data related to the types of data with uncertain predictions) or correct predictions (e.g., by alerting the user to regions that require manual review).

### 5.7. Analysis of Using Segmentation Masks for Food Composition Estimation

**Figure 9:**
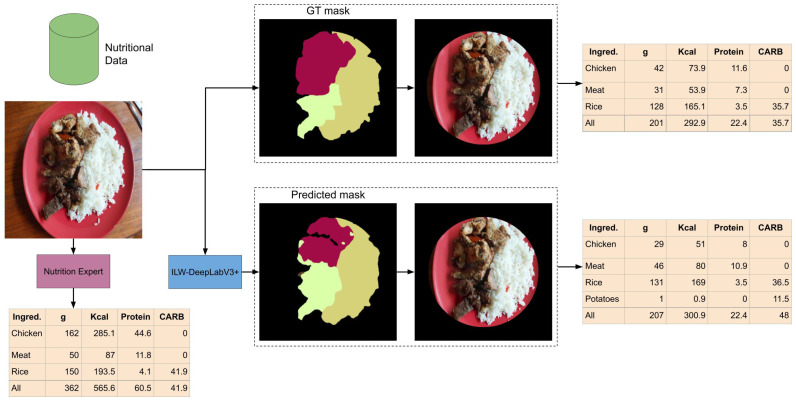
Example of nutrition values estimation from a semantic segmentation mask and standardized food composition data.

Figure 9 shows the results of estimating nutritional values from input images using the GT segmentation mask, the predicted segmentation mask, and the nutrition expert’s evaluation. In all cases, standardized food composition data were used. A straightforward approach is used to illustrate how food composition data can be estimated from segmentation masks. First, the background of the input image is removed by applying a circular mask that covers the food. This allows for the calculation of the total area where the food is placed, from which the proportional area occupied by the food on the plate is estimated. Second, for each predicted food item, its weight (in grams) is estimated based on the surface area it covers, using information from the atlas. Third, the energy (Kcal), grams of protein, and grams of available carbohydrates (CARB) are calculated based on the estimated weight of the food, using food composition data extracted from standardized data for each item.

By analyzing the estimated food composition, it can be observed that the results are very similar when using the predicted masks compared to the GT masks. This is interesting because the predicted mask does not fully overlap with the GT mask. There are some notable differences between them: overestimation of meat and underestimation of chicken, better estimation of the background (smaller error in the GT mask), and erroneous prediction of extra food (potatoes). This suggests that some food items, such as meat and chicken, could be grouped to simplify the problem’s complexity without significantly affecting the estimation accuracy. In contrast, when comparing the results of the segmentation masks with the assessment of a nutrition expert, the difference is more pronounced, especially in the estimation of grams of chicken. Although the food image may appear simple to analyze at first glance, it presents several challenges that highlight the limitations of segmentation, such as the volume of the food (e.g., a chicken wing placed on top of another) and occlusions (e.g., meat and chicken covering the rice). Therefore, to be used in a real application, it is necessary to consider a very controlled environment to ensure a more accurate estimation.

## 6. Discussion

The evaluation of ILW-DeepLabv3+ highlights its ability to achieve a strong balance between efficiency, segmentation accuracy, and computational cost, making it a compelling alternative to heavier architectures such as DeepLabv3+, FoodSAM, and BayesianGourmetNet. By significantly reducing the number of parameters and MACs, the model maintains a high mIoU while ensuring real-time feasibility, making it well suited for resource-constrained environments, such as mobile applications and embedded systems.

A key factor contributing to these improvements is the integration of attention mechanisms, which enhance feature extraction and spatial awareness. The results indicate that different attention modules impact segmentation accuracy in varying ways. For example, GAM achieves the highest mIoU on UECFoodPixComplete, while SE consistently performs well across multiple datasets due to its adaptability in feature recalibration. The experiments further confirm that all tested attention mechanisms improve performance, although each one introduces different computational trade-offs, with ECA and SIMAM offering gains while maintaining minimal parameter overhead. These findings reinforce the importance of carefully selecting attention modules to balance accuracy and computational efficiency in lightweight models.

In addition, a qualitative analysis of the segmentation results reveals both successful cases and limitations. Structured meals, such as those with clearly defined boundaries, exhibit highly accurate segmentation, closely matching the GT annotations. However, overlapping food items, sauces, and visually similar components result in segmentation challenges, occasionally leading to merged or misclassified regions. This issue is particularly evident in stews and mixed dishes. Addressing these segmentation difficulties remains an open challenge for future research.

Another important aspect of this work is the use of conformal prediction, which increases the interpretability of the results and allows us to leverage the estimated uncertainty for a deeper understanding of model errors. This approach not only helps identify regions where the model is less confident but also provides a way to improve the segmentation process by highlighting areas that may require further attention or correction.

Finally, a simple case study on estimating food composition data from food segmentation underscores the complexities of using segmentation for this purpose in an unconstrained environment. Several challenges must be addressed to ensure accurate estimations, including (1) handling overlapping food items, (2) ensuring robustness to random noise introduced during data acquisition, (3) collecting sufficient data to capture the variability of the target cuisine style, and (4) accurately estimating food size without a reference point, among others. Therefore, while semantic food segmentation provides valuable information for estimating food composition data and may be sufficient in highly controlled environments, it needs to be combined with other methods (e.g., volume estimation, depth estimation, etc.) to enhance the accuracy of the estimation in real-life environments.

### Limitations

The proposed ILW-DeepLabv3+ achieved remarkable results in the semantic food segmentation task in an efficient manner. However, we identified the following limitations that could be addressed as possible research directions:

- **Long-Tailed Food Segmentation Problem:** One of the key limitations identified is the long-tailed distribution of food categories in the dataset, where certain food items appear significantly more frequently than others. This imbalance negatively impacts segmentation performance, as the model tends to prioritize majority classes while failing to generalize to underrepresented food items. To address this issue, data augmentation using synthetic images can be employed to artificially increase the dataset size for minority classes. Techniques such as generative adversarial networks (GANs) or diffusion models can be used to generate realistic training samples for underrepresented images in general, improving their representation in the dataset. Additionally, loss weighting strategies (e.g., focal loss or class-balanced loss) can be incorporated during training to compensate for class imbalances.
- **Accuracy in Segmentation of Small Food Regions:** Another limitation identified is the segmentation of small food regions, such as dishes served far from the main content or vegetables cut into small pieces, among others. To address this issue, several techniques can be explored. The most straightforward approach is to use high-resolution inputs. However, additional improvements in computational efficiency must be made to counterbalance the increased resource demands associated with using larger images. On the other hand, using larger dilated convolutions or adding more layers to the pyramid pooling can enhance multi-scale feature learning, thereby improving the segmentation of objects of different sizes. Additionally, replacing cross-entropy loss with Dice loss can further enhance segmentation.

## 7. Conclusions

In this work, we proposed ILW-DeepLabv3+, a lightweight variant of DeepLabv3+ designed to improve semantic food segmentation while significantly reducing computational costs. By optimizing the encoder structure and incorporating attention mechanisms, our model strikes an effective balance between accuracy and efficiency, making it well suited for resource-constrained environments. The experimental results demonstrate that ILW-DeepLabv3+ achieves competitive or superior performance compared to state-of-the-art models while requiring substantially fewer parameters and MACs. Despite these advancements, challenges remain in segmenting overlapping food items and accurately estimating portion sizes, highlighting areas for further improvement. Additionally, our results emphasize the importance of high-quality annotated datasets, as well as the need for food composition and volumetric information, to enhance the practical applicability of segmentation models in dietary monitoring and health-related applications.

Future work will focus on further improving performance in lightweight semantic food segmentation by implementing additional architectural efficiency strategies and model compression techniques, such as pruning, quantization, and knowledge distillation. Particularly for architectural efficiency, we aim to design a hybrid CNN–Transformer architecture. Furthermore, advanced training strategies, such as data augmentation using generative AI and class-specific loss weighting, will be explored. We will also investigate multi-view and depth-based approaches to improve the accuracy of food composition data estimation.

## Figures and Tables

**Table 1:** Experimental results in terms of mIoU when integrating different attention mechanisms. The best and second-best results are in bold and underlined, respectively.

Attention Mechanism	UECFoodPixComplete	UNIMIB2016	MyFood	CamerFood10	SAI-ChileanFoodSeg
LW-DeepLabv3+	0.6439	0.7284	0.8137	0.8043	0.3205
CBAM [44]	0.6914	0.7924	0.8365	0.8249	**0.3486**
ECA [45]	0.6808	0.7985	0.8416	0.8268	0.3349
GAM [46]	**0.6985**	**0.8040**	0.8377	0.8269	0.3308
SE [47]	0.6926	0.7918	**0.8451**	**0.8469**	0.3466
SIMAM [14]	0.6851	0.7805	0.8425	0.8316	0.3457
TAM [48]	0.6917	0.7981	0.8415	0.8372	0.3445

**Table 2:** Increase in computational cost when integrating different attention mechanisms. Model parameters and MACs are calculated based on the number of classes in the UECFoodPixComplete dataset.

Attention Mechanism	Parameters	MACs (M)
LW-DeepLabv3+	6,793,767	2541.89
CBAM	+8290	+0.38
ECA	+5	+0.27
GAM	+32,870	+33.67
SE	+8192	+0.28
SIMAM	+0	+0
TAM	+200	+1.68

**Table 3:** Performance of ILW-DeepLabV3+ on the UECFoodPixComplete and UNIMIB2016 datasets using different learning rates. The results of the last epoch are shown in parentheses.

Learning Rate	UECFoodPixComplete	UNIMIB2016
0.001	0.6246 (0.6193)	0.0507 (0.0507)
0.005	0.6792 (0.6771)	0.5275 (0.5275)
0.010	0.6926 (0.6880)	0.6901 (0.6901)
0.050	0.6451 (0.6451)	0.7918 (0.7891)
0.100	0.5607 (0.5541)	0.8124 (0.8080)

**Table 4:** Performance evaluation of the proposed method on the UECFoodPixComplete dataset after each optimization.

	Optimization			Performance Metric				
Method	Backbone	Neck	Head	Parameters	MACs	GPU (s)	CPU (s)	mIoU
DeepLabV3+ (ResNet101)	✗	✗	✗	45.70M	57.13G	0.0238	0.3016	0.6636
DeepLabV3+ (EfficientNet-B1)	✓	✗	✗	7.44M	3.14G	0.0145	0.1683	0.6864
LW-DeepLabV3+	✓	✓	✗	6.79M	2.54G	0.0142	0.1644	0.6439
ILW-DeepLabV3+	✓	✓	✓	6.80M	2.54G	0.0142	0.1642	0.6926

**Table 5:** Evaluation of different lightweight backbones in the proposed method. The results of the last epoch are shown in parentheses.

	UECFoodPixComplete		CamerFood10	
Lightweight Backbone	mIoU	CPU (s)	mIoU	CPU (s)
ShuffleNetV2+	0.2901 (0.2713)	0.0760	0.5876 (0.5272)	0.0499
MobileNetv3-Large	0.6133 (0.6098)	0.0897	0.8007 (0.7933)	0.0633
EfficientNet-B1	0.6926 (0.6880)	0.1642	0.8469 (0.8288)	0.1388

**Table 6:** Semantic food segmentation performance on the UECFoodPixComplete dataset. (+) used in GourmentNet indicates that the inference time is at least as indicated and increases as a function of the forward passes considered in this method.

Method	mIoU	Parameters (M)	MACs (G)	GPU (s)	CPU (s)
YOLACT [28]	0.5485	42.34 *	42.07 *	-	-
DeepLabv3+ [27]	0.5550	45.70	57.12	0.0238	0.3016
GourmetNet [30]	0.6513	91.82	49.88	0.0202	0.2420
FoodSAM [50]	0.6614	636.00	1016.72	-	-
BayesianGourmetNet [32]	0.6616	91.82	49.88	0.0202 (+)	0.2420 (+)
CANet [31]	0.6890	40.10	127.72	-	-
FDSNET [4]	0.7589	101.93	91.37	-	-
LW-DeepLabv3+ [16]	0.6522	6.79	2.54	0.0142	0.1644
ILW-DeepLabv3+	0.6926	6.80	2.54	0.0142	0.1642

* Only the backbone network is considered for estimating Parameters and MACs.

**Table 7:** Semantic food segmentation performance on the UNIMIB2016 dataset. (+) used in GourmentNet indicates that the inference time is at least as indicated and increases as a function of the forward passes considered in this method.

Method	mIoU	Parameters (M)	MACs (G)	GPU (s)	CPU (s)
DeepLabv2 [25]	0.4300	42.5 *	52.19 *	-	-
SegNet [26]	0.4400	42.5 *	52.19 *	-	-
GourmetNet [30]	0.7179	91.79	48.80	0.0332	0.4071
BayesianGourmetNet [32]	0.8076	91.79	48.80	0.0332 (+)	0.4071 (+)
LW-DeepLabv3+ [16]	0.7449	6.79	2.34	0.0141	0.1564
ILW-DeepLabv3+	0.7918	6.79	2.34	0.0141	0.1570

* Only the backbone network is considered for estimating Parameters and MACs.

**Table 8:** Semantic food segmentation performance on the MyFood dataset.

Method	mIoU	Parameters (M)	MACs (G)	GPU (s)	CPU (s)
Mid-DeepLabv3+ [11]	0.6923	10.41	40.96	0.0178	0.3130
CDPN [29]	0.7700	42.52 *	41.09 *	-	-
ILW-DeepLabv3+	0.8451	6.77	1.89	0.0137	0.1388

* Only the backbone network is considered for estimating parameters and MACs.

**Table 9:** Semantic food segmentation performance on the CamerFood10 dataset.

Method	mIoU	Parameters (M)	MACs (G)	GPU (s)	CPU (s)
Mid-DeepLabv3+ [11]	0.6520	10.41	40.96	0.0178	0.3130
ILW-DeepLabv3+	0.8469	6.77	1.89	0.0137	0.1388

## Data Availability

The UECFOODPIX, UNIMIB2016, MyFood, and CamerFood10 datasets are publicly available at the following links: https://mm.cs.uec.ac.jp/uecfoodpix/ (accessed on 17 February 2025), http://www.ivl.disco.unimib.it/activities/food-recognition/ (accessed on 17 February 2025), https://drive.google.com/drive/u/1/folders/1MugfmVehtIjjyqtphs-4u0GksuHy3Vjz (accessed on 17 February 2025), and https://drive.google.com/drive/u/1/folders/1MugfmVehtIjjyqtphs-4u0GksuHy3Vjz (accessed on 17 February 2025). The SAI-ChileanFoodSeg dataset is available on request from the corresponding author because the data are part of an ongoing study.
